# Supplementary material for: Identification and Elimination of the Clinically Relevant Multi-Resistant Environmental Bacteria Ralstonia insidiosa in Primary Cell Culture
Source: Microorganisms. 2020 Oct 17;8(10):1599. doi: 10.3390/microorganisms8101599 (PMC7603027; doi:10.3390/microorganisms8101599)
Supplement: Supplementary file 1 [file microorganisms-08-01599-s001.pdf]

Table S1. Antimicrobial resistance genes derived from the draft genome of our *Ralstonia insidiosa* isolate.

GENE	%COVERAGE	%IDENTITY	DATABASE	PRODUCT	RESISTANCE
ceoA	78.65	70.31	card	ceoA is a periplasmic linker subunit of the CeoAB-OpcM efflux pump	aminoglycoside; fluoroquinolone
ceoB	99.25	79.27	card	ceoB is a cytoplasmic membrane component of the CeoAB-OpcM efflux pump	aminoglycoside; fluoroquinolone
MuxB	97.09	75.47	card	MuxB is one of the two necessary RND components in the Pseudomonas aeruginosa efflux pump system MuxABC-OpmB.	aminocoumarin; macrolide; monobactam; tetracycline
MuxC	96.59	72.08	card	MuxC is one of the two necessary RND components of the MuxABC-OpmB efflux pumps system in Pseudomonas aeruginosa.	aminocoumarin; macrolide; monobactam; tetracycline
blaOXA-573	100.00	95.34	ncbi	OXA-60 family carbapenem-hydrolyzing class D beta-lactamase OXA-573	CARBAPENEM [a]
blaOXA-574	100.00	95.12	ncbi	OXA-22 family class D beta-lactamase OXA-574	BETA-LACTAM
mdsB	98.52	75.38	card	MdsB is the inner membrane transporter of the multidrug and metal efflux complex MdsABC.	carbapenem; cephalosporin; cephamycin; monobactam; penam; penem; phenicol
golS	81.08	75.86	card	GolS is a regulator activated by the presence of golD and promotes the expression of the MdsABC efflux pump.	carbapenem; cephalosporin; cephamycin; monobactam; penam; penem; phenicol
AxyY	97.48	73.60	card	AxyY is the periplasmic adaptor protein of the AxyXY-OprZ efflux pump system in Achromobacter spp.	aminoglycoside; cephalosporin; fluoroquinolone; macrolide

[a] OXA-60-like carbapenemase only exhibit hydrolysis of imipenem and not meropenem [1,2].

1. Girlich, D.; Naas, T.; Nordmann, P. Oxa-60, a chromosomal, inducible, and imipenem-hydrolyzing class d beta-lactamase from ralstonia pickettii. *Antimicrob Agents Chemother* **2004**, *48*, 4217-4225.
2. Walther-Rasmussen, J.; Hoiby, N. Oxa-type carbapenemases. *J Antimicrob Chemother* **2006**, *57*, 373-383.

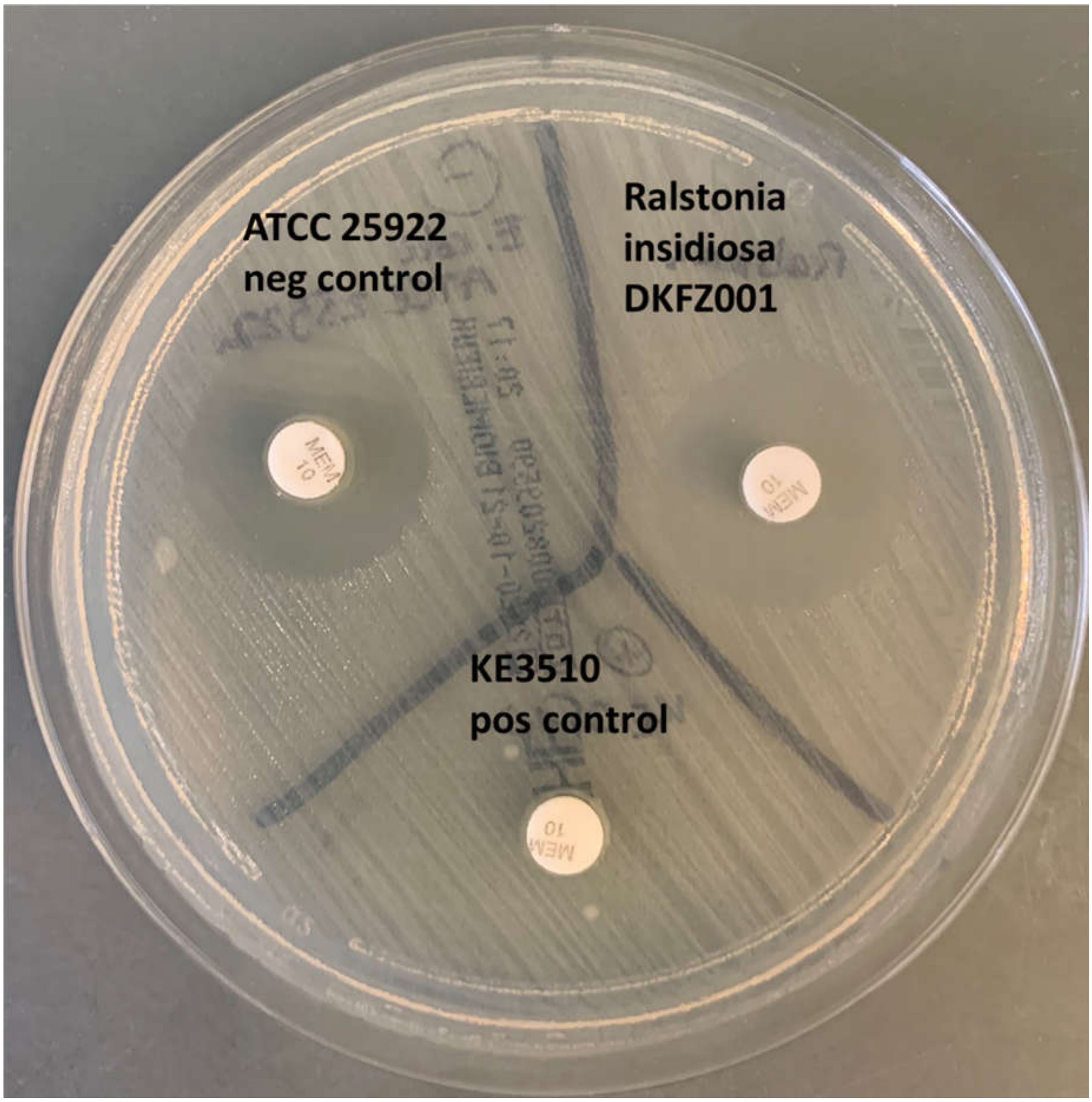

Figure S1: Our *Ralstonia insidiosa* (DKFZ001) isolate did not hydrolyze meropenem (top right) as indicated by the similar zone of inhibition comparable to the carbapenemase-negative multi-susceptible *E. coli* ATCC®25922 (top left). The positive control (carbapenemase-producing *C. amalonaticus* KE3510) was able to fully hydrolyze meropenem, resulting in an absence of an inhibition zone (bottom).
